# Acute Anaerobic Exercise Affects the Secretion of Asprosin, Irisin, and Other Cytokines – A Comparison Between Sexes

**DOI:** 10.3389/fphys.2018.01782

**Published:** 2018-12-10

**Authors:** Magdalena Wiecek, Jadwiga Szymura, Marcin Maciejczyk, Malgorzata Kantorowicz, Zbigniew Szygula

**Affiliations:** ^1^Department of Physiology and Biochemistry, Faculty of Physical Education and Sport, University of Physical Education in Krakow, Kraków, Poland; ^2^Department of Clinical Rehabilitation, Faculty of Motor Rehabilitation, University of Physical Education in Krakow, Kraków, Poland; ^3^Faculty of Physical Education and Sport, University of Physical Education in Krakow, Kraków, Poland; ^4^Department of Physiotherapy, State University of Applied Sciences in Nowy Sacz, Nowy Sącz, Poland

**Keywords:** asprosin, irisin, adipocytokines, anaerobic exercise, sex differences

## Abstract

**Objective:** The new adipokine, which is asprosin, affects glucose release from the liver to the blood, and thus, influences exercise metabolism. This is the first study assessing whether single anaerobic exercise affects asprosin secretion in women and men.

**Methods:** 10 men and 10 women (aged 21.64 ± 1.22 and 22.64 ± 1.49, respectively) performed a single 20-s bicycle sprint. Blood samples were collected before exercise and in the 3′, 15′, 30′, and 60′ of recovery, and 24 h after competition.

**Results:** Only in women did asprosin (*P* = 0.001) (15′, 30′, 60′, and 24 h after exercise) and irisin (*P* < 0.001) (15′, 30′, and 60′) concentrations increase. Leptin, however, decreased (*P* = 0.001) at 3′, 15′, and 30′ in women. There was an increase in interleukin-6 (*P* < 0.001) at 3′, 15′, 30′, and 60′ of recovery in men, at 15′, 30′, 60′, and 24 h of recovery in women, along with a simultaneous decrease in interleukin-1β (*P* < 0.001) at 15′, 30′, and 60′ of recovery in men, and at 15′ and 30′ of recovery in women (*r* = -0.35, *P* < 0.001). There was a positive correlation between asprosin and adiponectin and a negative one between asprosin and leptin. The increase in irisin concentration at 30′ of recovery was positively correlated with the increase in asprosin concentration and percentage fat content, while being negatively correlated with total and lean body mass (LBM).

**Conclusion:** The single anaerobic effort induced an increase in asprosin and irisin secretion while reducing leptin secretion in women. Adipocytokine concentration changes are inter-related. Regardless of sex, anaerobic efforts induce anti-inflammatory effects.

## Introduction

Short, dynamic anaerobic exercise, such as a 20-s bicycle sprint, induces enormous multidirectional biochemical changes, including metabolic, hormonal, and immunological ones. It has been shown that, both single anaerobic exercises (Wiecek et al., 2015, 2017, 2018), as well as a series of anaerobic exercises (Bogdanis et al., 2013), induce oxidative stress immediately after the exercise and also during the early period of recovery, and even 24 h after its completing. The effect of anaerobic exercise is also a significant increase in the concentration of IL-6 and the number of neutrophils and monocytes, the number of which was also increased 24 h after completion of the effort (Meyer et al., 2001). A high correlation between stress indicators of oxidative stress such as hydrogen peroxide (H_2_O_2_) and 8-isoprostanes as well as the number of neutrophils and the concentration of anti-inflammatory interleukins (IL-6 and IL-10) was noted, and at the same time, a positive correlation between exercise-induced level of nitric oxide (nitro-oxidative stress index) and pro-inflammatory IL-1β and TNF-α (Petersen and Pedersen, 2005; Zembron-Lacny et al., 2010). It has also been demonstrated that a single anaerobic exercise increases the nuclear factor κB (NF-κB) activity in the nuclei of peripheral blood mononuclear cells, which is accompanied by the degradation of its inhibitor (IκB) (Cuevas et al., 2005). NF-κB is activated by reactive oxygen species (He et al., 2016; Ji et al., 2016), and mediates pro- and anti-inflammatory processes (Lawrence, 2009), thereby influencing the expression of interleukins as well as adipocytokines and myokines (Zamboni et al., 2007; Piotrowska et al., 2008; Al-Anazi et al., 2018). Therefore, an anaerobic effort may cause an increase in adipocytokine and myokine concentration in the blood which are involved in the regulation of resting and exercise-related metabolic processes (Bostrom et al., 2012; Li et al., 2017). They affect energy balance and anti-inflammatory activity (Cao, 2014; Li et al., 2017).

Romere et al. (2016) discovered asprosin, a new peptide hormone involved in regulating glucose level. Asprosin is a C-terminal cleavage product of profibrillin (encoded by *FBN1*), it is secreted into the bloodstream mainly by the cells of the white adipose tissues. *Via* the G-protein/cAMP-protein kinase A pathway, it regulates the release of glucose from hepatocytes. Higher levels of asprosin were observed in obese subjects and in those with type 2 diabetes (Romere et al., 2016; Zhang et al., 2017). There was a positive correlation between fasting glucose and triacyloglycerole concentration as well as asprosin concentration in type 2 diabetic and healthy patients (Zhang et al., 2017). In the case of losing asprosin function (genetic or immunological modification), glucose and insulin levels are drastically reduced as a result of the decrease in hepatic glucose release (Romere et al., 2016). The expression of *FBN1* mRNA was also found in other tissues, including the skeletal muscles (Romere et al., 2016). This may indicate the potential role of this mediator in releasing glucose from muscle glycogen during physical exercise, mainly of higher intensity (anaerobic exercise), when the dominant substrate is glucose (Beneke et al., 2002); however, there are no such studies that would confirm this.

Other hormones may also affect anaerobic metabolism during high-intensity exercise. Irisin is secreted by myocytes (Bostrom et al., 2012) as well as adipocytes (Roca-Rivada et al., 2013), and stimulates glycolysis, affecting the increase of lactate synthesis (Huh et al., 2014). This hormone also effect glucose homeostasis *via* upregulation of glucose uptake by cells and glycogenolysis, and reduction of gluconeogenesis, adipogenesis, and lipid accumulation (Liu et al., 2015; Xiong et al., 2015; Gao et al., 2016; Mo et al., 2016). It is known, that in sprinters, the blood concentration of irisin is higher after high-intensity exercise (Pukajlo et al., 2015). It was found that the level of irisin in the blood negatively correlates with the concentration of leptin (Palacios-Gonzalez et al., 2015), while positively correlating with adiponectin concentration (Nigro et al., 2017). Adiponectin increases insulin sensitivity of myocytes and hepatocytes by inhibiting hepatic gluconeogenesis and increasing glucose uptake in the skeletal muscles. The level of adiponectin was elevated after anaerobic exercise (Ozturk et al., 2016), increasing the consumption of glucose (as an energy substrate) and lactate production (Yamauchi et al., 2002; Fernandez-Sanchez et al., 2011; Ouchi et al., 2011; Coelho et al., 2013). The relationship between secretion of adipocytokines and interleukins was observed (Yamauchi et al., 2002; Gnacinska et al., 2009; Fernandez-Sanchez et al., 2011).

To our knowledge, this is the first study determining whether there is a change in the concentration of asprosin in the blood of women and men as a result of a single anaerobic energy-based effort. The aim of our research is also to determine the effects of a single anaerobic exercise on the secretion of other adipocytokines/myokines such as irisin, as well as pro-inflammatory and anti-inflammatory interleukins and the mutual relationships between their secretions.

In order to determine the effect of anaerobic exercise on changes in adipocytokine/myokine concentrations in the blood, we conducted research involving young women and men who performed a 20-s supramaximal cycling sprint. In the blood, we marked the level of hormones, the function of which is related to glucose metabolism, that is the main substrate for ATP resynthesis in such efforts (Morales-Alamo and Calbet, 2014), i.e., asprosin, irisin, adiponectin, and leptin, as well as interleukins (IL-6, IL-1β, and IL-10), which, among others, are also secreted by muscle cells and adipocytes (Zembron-Lacny and Ostapiuk-Karolczuk, 2008; Li et al., 2017). Marking was conducted several times during the first hour of recovery and 24 h after completing the anaerobic exercise.

We hypothesize that a single anaerobic effort affects the secretion of adipocytokines/myokines, causing, among others, an increase in asprosin and irisin levels in the blood of both women and men, while having anti-inflammatory effects.

## Materials and Methods

The research was carried out in accordance with the Declaration of Helsinki. All volunteers provided written consent for participation after becoming acquainted with the procedures, purpose, and research plan. The approval of the local Bioethical Committee (81/KBL/OIL/2013) was obtained for conducting research according to the presented methodology.

### Participants

The study included 10 men and 10 Caucasian women aged 21.64 ± 1.22 and 22.64 ± 1.49 years, respectively. All of them were non-smokers, and were physically active, but did not perform any sports disciplines. The participants’ physical activity (7.4 ± 2.9, 2.2 ± 1.1, and 1.5 ± 1.3 h/week, respectively, moderate – four METs, hard – six METs, and very hard intensity – 10 METs) was assessed using the 7-day PAR questionnaire (Sarkin et al., 1997). None of the participants suffered from chronic diseases and used dietary supplements or vegetarian/vegan diets.

#### Somatic Build

These were individuals with normal body mass (BM) and composition, and normal metabolic profile. BM, body mass index (BMI), percentage of body fat (%FAT), and lean body mass (LBM) significantly differed in both groups and totaled 77.13 ± 8.56 kg, 23.71 ± 1.58 kg/m^2^, 18.36 ± 2.99%, and 62.95 ± 7.29 kg and 59.75 ± 6.49 kg (*P* < 0.001), 21.50 ± 1.84 kg/m^2^ (*P* = 0.009), 24.20 ± 1.60% (*P* < 0.001), and 45.15 ± 4.12 kg (*P* < 0.001) for men and women, respectively.

Body height was measured using a Martin-type anthropometer to the nearest 1 mm (Poland). BM and body composition were determined using a multi-frequency (5, 50, and 250 kHz), eight-electrode analyzer (Jawon IOI-353 Body Composition Analyzer, Korea), based on bioelectrical impedance analysis (BIA).

#### State of Health

The participants’ state of health was confirmed by medical examination (medical history, physical examination, ECG, morphology, fasting state glycemia and lipid profile, percentage of glycated hemoglobin). The results of the analyses are presented in Table 1. No medical contraindications to extreme endurance efforts were found. Women had a regular menstrual cycle (27–32 days) and did not use any hormonal contraceptives for at least 3 months prior to the trial.

**Table 1:** The results of the medical qualifications of study participants.

Variables	Men (*n* = 10)	Women (*n* = 10)	*P*-value
Erythrocytes (10^6^/μL)	5.20 ± 0.26	4.42 ± 0.37	<0.001
Hemoglobin (g/dL)	15.70 ± 0.77	13.01 ± 1.16	<0.001
Hematocrit (%)	45.33 ± 1.48	38.64 ± 3.17	<0.01
ESR (mm/h)	2.50 (2.0–4.0)	4.00 (3.0–5.0)	(0.123)
Platelets (10^3^/μL)	252.80 ± 44.92	235.00 (207.0–257.0)	(0.353)
Leukocytes (10^3^/μL)	5.68 ± 1.13	5.75 (5.0–6.2)	(0.971)
Neutrophils (%)	56.66 ± 6.46	57.05 (55.0–59.3)	(0.631)
Lymphocytes (%)	33.61 ± 6.86	32.05 (29.5–34.2)	(0.579)
Monocytes (%)	7.38 ± 1.93	7.62 ± 1.98	0.787
Eosinophils (%)	1.86 ± 0.97	2.23 ± 1.21	0.460
Basophils (%)	0.30 (0.2–0.5)	0.40 ± 0.16	(0.393)
Glucose (mg/dL)	74.21 ± 6.73	79.92 ± 9.27	0.133
HbA1c (%)	5.00 ± 0.44	4.90 ± 0.27	0.546
Total cholesterol (mg/dL)	182.83 ± 27.13	167.56 ± 14.75	0.135
HDL (mg/dL)	53.94 (51.82–59.17)	60.83 ± 6.48	(0.123)
LDL (mg/dL)	108.43 ± 20.06	94.90 ± 14.21	0.099
Triglycerides (mg/dL)	83.96 ± 22.41	59.52 ± 15.16	0.010


*Values are means ± SD or median (IQR); *P* < 0.05, significant differences women vs. men (*t*-test or Mann–Whitney *U* test); IQR, interquartile range; ESR, erythrocyte sedimentation rate; HbA1c, glycated hemoglobin; HDL, high-density lipoproteins; LDL, low-density lipoprotein.*

#### Physical Fitness

In previous studies, in the graded test, the following were determined for each of the volunteers: maximum heart rate – HR_max_ and oxygen efficiency by measuring maximal oxygen consumption per minute – VO_2max_; and during the sprint: maximum (MAP) and mean (MP) anaerobic power of the lower limbs were measured (Wiecek et al., 2015). HR_max_ was similar in both groups (*P* = 0.354) and was 201.0 ± 11.1 and 196.3 ± 10.1 b/min, in men and women, respectively. VO_2max_ was significantly higher (*P* < 0.001) in men (55.61 ± 5.63 mL/kg), compared to women (44.75 ± 3.43 mL/kg). MAP and MP were also higher in the group of men than women and were as follows: 11.28 ± 0.82 and 8.59 ± 0.44 W/kg (*P* < 0.001) for MAP and: 9.41 ± 0.62 and 7.33 ± 0.49 W/kg (*P* < 0.001) for MP, respectively.

### Study Design

Each participant performed a single 20-s bicycle sprint (824E Monark, Sweden). Venous and capillary blood was collected before the exercise (0′) and five times during the recovery period: third minute (3′), 15th minute (15′), 30th minute (30′), 60th minute (60′), and 24 hours (24 h) following the test. Asprosin, irisin, adiponectin, leptin, glucose, and lactate were determined as well as interleukin concentration: IL-1β, IL-6, and IL-10. Hemoglobin and hematocrit were determined to calculate changes in plasma volume.

### Cycle-Sprint

Execution of the bicycle sprint has been previously described (Wiecek et al., 2015). First, there was a warm-up lasting 4 min, based on pedaling at a rate of 60 revolutions per minute with a load of 90 W for men and 60 W for women. At the end of the second and fourth minutes of the warm-up, the maximum acceleration of pedaling cadence lasted for 5 s. After the warm up, there was a 4-min break and then a bicycle sprint. The cycling sprint was performed in a seated position, with a constant, individually selected load of 5.79 ± 0.64 kg for men and 3.88 ± 0.42 kg for women (*P* < 0.001), which corresponded to 0.075 × BM and 0.065 × BM. The test effort consisted in obtaining the maximum cadence of pedaling as quickly as possible and maintaining it for 20 s. The participants started the sprint after hearing the 3-2-1-GO! command and were vigorously encouraged. After 20 s, the load was removed and the subjects pedaled at 60 rpm for 3 min.

The efforts were carried out under the supervision of a physician. The study was conducted from October to December in Poland, the external temperature was about 15°C to -5°C. Due to the possible impact of the time of day and ambient temperature on muscular power (Racinais et al., 2004; Tyka et al., 2009), laboratory conditions were unified and controlled. All efforts were performed in thermally neutral conditions (20–22°C), in the morning (9.00–11.00 a.m.), after 8 h of sleep, 2 h after a light breakfast. The women performed the effort between the sixth and ninth days of the follicular phase. The first day of menstruation was the first day of the follicular phase. The participants did not perform intense physical efforts for 7 days preceding and on the day of the exercise test as well as for 24 h after its completion. The participants did not drink any beverages directly before the sprint or during the recovery period, they also did not consume alcoholic, caffeine- or taurine-containing beverages 24 h before and after the end of their workout.

### Biochemical Analysis

Blood plasma concentrations of adipocytokines: asprosin, irisin, adiponectin, leptin, and serum concentrations of interleukins: IL-1β, IL-6, and IL-10 were determined using the enzyme immunoassay (ELISA) method in accordance with the manufacturer’s manuals.

The asprosin concentration was determined using the Human Asprosin ELISA KIT SK00229-06 (Aviscera Bioscience, Inc., United States). The concentration of irisin was determined using the Irisin/FNDC5 EK-067-16 (Phoenix Pharmaceuticals, Inc., United States). Adiponectin concentration was determined with the high-sensitivity Human Adiponectin Elisa RD191023100, whereas leptin concentration was measured with the Human Leptin Elisa RD191001100 test (BioVendor, the Czechia). The detection range for asprosin was 1–32 nmol/L, for irisin 0–100 ng/mL, for adiponectin 2–150 ng/mL, and for leptin 0–50 ng/mL. Intra-assay CV for asprosin was <8%, for adiponectin it totaled 4.4%, for leptin 7.6%, and the inter-assay CV for asprosin was <12%, for adiponectin it equaled 6.2%, while for leptin, it was 6.7%.

IL-1β, IL-6, and IL-10 concentrations were determined using R&D Systems (United States). Test sensitivity for IL-1β was 0.057 pg/mL (HSLB00C), for IL-6, it totaled 0.039 pg/mL (HS600B), and for IL-10, it was 0.09 pg/mL (HS100C). Intra-assay CV for IL-1β equaled <10.2%, for IL-6 <7.8%, for IL-10 <9.4%, while the inter-assay CV for IL-1β was <10.4%, for IL-6 <7.2%, and for IL-10 <8.5%.

The concentrations of lactate and glucose were determined in the capillary blood plasma *via* the colorimetric enzymatic method according to the manufacturer’s instructions using LC2389 and GL2623 (Randox, United Kingdom), for which the absorbance and concentration dependence was maintained at 19.7 and 22.2 mmol/L, respectively.

The hemoglobin concentration was determined with the cyanide-methemoglobin method (Drabkin’s reagent, Poland), while hematocrit with the micro-hematocrit method in triplicate, and the mean was calculated. Knowing hemoglobin and hematocrit levels, which were determined in whole venous blood immediately after collection, plasma volume changes were calculated (Dill and Costill, 1974; Harrison et al., 1982) and then, the concentration of all marked biochemical markers were adjusted by these changes (Kraemer and Brown, 1986).

### Diet Analysis

Because the varied diet used by volunteers could affect the anaerobic power (Wroble et al., 2018) and the level of adipocytokines in the blood (Reis et al., 2010; de Luis et al., 2016; Huang et al., 2017), the meal guidelines were proposed in our studies by a dietician taking the age, sex, and physical activity of the participants into account (2800 kcal/day for man, 2000 kcal/day for women; carbohydrates 55%, fat 30%, and proteins 15%) (Jarosz, 2017). The diet was assessed based on the analysis of dietary diaries filled in by the participants using the product and food photography album (Szponar et al., 2012) during the 7 days preceding the cycling sprint. Analysis was carried out using the Diet 5.0 computer program (Institute of Food and Nutrition, Poland).

### Statistical Analysis

Data distribution was checked using the Shapiro–Wilk test. Significance of differences between sexes for single measurements was tested with the *t* test for independent samples or the Mann–Whitney *U* test. The Friedman ANOVA test for multiple measurements and the Wilcoxon test were used to assess the effect of exercise on changes in biochemical markers. The results for men and women were compared with the Mann–Whitney *U* test. The Spearman correlation coefficient was calculated. For all variables, differences at the level of *P* < 0.05 were considered statistically significant. Data are presented as mean ± SD (standard deviation) or median and IQR (interquartile range). The calculations were performed using the Statistica 12 program (Stat-Soft, Inc., the United States).

## Results

### The Effects of Anaerobic Exercise on the Level of Biochemical Markers

#### Lactate

In both groups, lactate concentration at 3′, 15′, 30′, and 60′ after the exercise was significantly higher than at baseline (*P* < 0.01). In women, the highest lactate concentration was at 3′, and in men, 15′ after the exercise (Table 2).

**Table 2:** The effects of anaerobic exercise (bicycle sprint) on blood lactate and glucose levels in men (*n* = 10) and women (*n* = 10).

		Exercise value						Friedman’s ANOVA
	
Variable	Sex	0′	3′	15′	30′	60′	24 h	*P*-value
Lactate (mmol/L)	Men	1.06 ± 0.37	9.03 ± 1.80^∗^	9.18 ± 0.78^∗^	6.91 ± 0.86^∗^	3.36 ± 0.98*,#	1.56 ± 1.18	<0.001
	Women	1.00 ± 0.46	7.83 ± 2.32^∗^	7.67 ± 2.33^∗^	5.46 ± 2.17^∗^	2.33 ± 1.48^∗^	1.00 ± 0.28	<0.001
Glucose (mmol/L)	Men	4.84 ± 0.71	4.22 ± 1.05	4.07 ± 0.97	4.45 ± 0.99#	4.80 ± 2.57	4.49 ± 0.79	0.255
	Women	4.72 ± 1.04	4.36 ± 1.01	4.12 ± 0.77	3.37 ± 0.61^∗^	4.07 ± 0.80	4.18 ± 1.32	0.047


*Value are means ± SD; Friedman’s ANOVA *P*-value < 0.05 – significant effect of exercise, ^∗^significant differences vs. baseline (*P* < 0.05); ^#^significant differences men vs. women (*P* < 0.05).*

#### Glucose

In women, 30′ after exercise, the glucose concentration was significantly lower than at baseline (*P* = 0.047). In men, there were no changes in glucose levels caused by exercise (Table 2).

#### Adipocytokines

The asprosin concentration in women at 3′ after exercise was comparable (*P* > 0.05) to baseline (0′), and then increased, attaining its highest value at 30′, which was significantly higher compared to the value at 3′ (*P* = 0.009) and 15′ (*P* = 0.017). Asprosin concentration at 60′ was still significantly higher compared to 3′ (*P* = 0.005) and 15′ (*P* = 0.047). In men, asprosin concentration did not change after the exercise (*P* = 0.312) (Table 3).

**Table 3:** The impact of anaerobic exercise (bicycle sprint) on adipocytokine level in the blood of men (*n* = 10) and women (*n* = 10).

		Exercise value						Friedman’s ANOVA
		
Variable	Sex	0′	3′	15′	30′	60′	24 h	*P*-value
Asprosin (nmol/L)	Men	5.94 ± 3.04	6.28 ± 3.85	5.87 ± 3.35	6.61 ± 4.47	6.04 ± 2.65	6.33 ± 3.45	0.312
	Women	4.02 ± 0.49	3.70 ± 0.70	4.05 ± 0.70^3′^	4.71 ± 1.70^3′,15′^	4.39 ± 0.83^3′,15′^	4.11 ± 0.98^30′^	0.001
Irisin (μg/mL)	Men	0.70 ± 0.49	0.74 ± 0.27	0.64 ± 0.28#	0.90 ± 0.37#	0.97 ± 0.35#	0.83 ± 0.44	0.187
	Women	0.83 ± 0.95	0.72 ± 0.93	2.12 ± 1.41^∗^	2.10 ± 0.71^∗^	1.63 ± 0.91^∗^	0.79 ± 0.85	<0.001
Adiponectin (μg/mL)	Men	9.23 ± 3.31	8.23 ± 2.19	9.52 ± 3.02	9.15 ± 4.06	8.88 ± 3.20	9.64 ± 3.20	0.141
	Women	9.31 ± 3.84	8.63 ± 3.45	9.26 ± 3.24	9.14 ± 3.99	9.14 ± 3.73	8.48 ± 3.57	0.353
Leptin (ng/mL)	Men	1.27 ± 1.02#	1.16 ± 1.00#	1.11 ± 1.12#	1.67 ± 1.86#	1.89 ± 2.32#	1.55 ± 1.47#	0.080
	Women	7.09 ± 3.93	5.57 ± 3.05^∗^	5.74 ± 3.12^∗^	6.13 ± 3.12^∗^	6.45 ± 3.35	5.90 ± 4.32	0.001


*Value are means ± SD; Friedman’s ANOVA *P*-value <0.05 – significant effect of exercise, ^∗^significant differences vs. baseline (*P* < 0.05); ^#^significant differences men vs. women (*P* < 0.05), ^*3*′ ,*15*′ ,*30*′^, significant differences vs. 3′, 15′, or 30′ (*P* < 0.05).*

In women, irisin concentration 15′, 30′, and 60′ after the exercise was significantly higher than at baseline (*P* < 0.001). In men, the concentration of irisin did not change (*P* = 0.187) (Table 3).

The concentration of adiponectin did not change in women (*P* = 0.353) or men (*P* = 0.141) after exercise (Table 3).

In women, the plasma concentration of leptin at 3′, 15′, and 30′ after exercise was significantly lower (*P* = 0.001) than at baseline. In men, the concentration of leptin following exercise did not change (*P* = 0.080) (Table 3).

#### Interleukins

In women, a significant increase in IL-6 concentration was found after exercise in the blood serum (*P* < 0.001) by 0.58 ± 0.14 pg/mL at 15′, by 0.70 ± 0.14 pg/mL at 30′, by 0.78 ± 0.12 pg/mL at 60′, and by 0.25 ± 0.11 pg/mL 24 h after the exercise. In the group of men, the serum concentration of IL-6 was significantly higher (*P* < 0.001) than the baseline by 0.31 ± 0.12 pg/mL at 15′, by 0.49 ± 0.12 pg/mL at 30′, and by 0.48 ± 0.10 pg/mL at 60′ following exercise (Table 4).

**Table 4:** The impact of anaerobic exercise (bicycle sprint) on the level of interleukins in the blood of men (*n* = 10) and women (*n* = 10).

		Exercise level						Friedman’s ANOVA
		
Variable (pg/mL)	Sex	0′	3′	15′	30′	60′	24 h	*P*-value
IL-6	Men	0.14 ± 0.07	0.17 ± 0.06^∗^	0.45 ± 0.35^∗^	0.63 ± 0.39^∗^	0.62 ± 0.31*,#	0.34 ± 0.24	<0.001
	Women	0.20 ± 0.19	0.19 ± 0.12	0.78 ± 0.44^∗^	0.90 ± 0.38^∗^	0.98 ± 0.35^∗^	0.45 ± 0.35^∗^	<0.001
IL-1β	Men	1.12 ± 0.20	0.98 ± 0.16	0.80 ± 0.22^∗^	0.85 ± 0.17^∗^	0.90 ± 0.19^∗^	1.17 ± 0.30	0.001
	Women	1.20 ± 0.25	1.15 ± 0.33	0.86 ± 0.14^∗^	0.88 ± 0.17^∗^	0.90 ± 0.35	1.11 ± 0.24	0.016
IL-10	Men	2.58 ± 1.24	2.34 ± 1.74	1.70 ± 0.98	2.18 ± 0.97	2.20 ± 1.05	2.51 ± 1.44	0.549
	Women	2.83 ± 1.22	2.69 ± 1.02	2.14 ± 0.90	2.33 ± 0.95	2.33 ± 2.26	3.08 ± 0.28	0.525


*Value are means ± SD; Friedman’s ANOVA *P*-value <0.05 – significant effect of exercise, ^∗^significant differences vs. baseline (*P* < 0.05); ^#^significant differences men vs. women (*P* < 0.05).*

In women, IL-1β was significantly lower after exercise than at baseline (*P* = 0.016) by 0.34 ± 0.12 pg/mL and by 0.32 ± 0.11 pg/mL at 15′ and 30′, respectively. IL-1β concentration in men significantly reduced (*P* = 0.001) by 0.33 ± 0.11 pg/mL at 15′, by 0.27 ± 0.08 pg/mL at 30′, and by 0.22 ± 0.09 pg/mL 60′ after exercise (Table 4).

In none of the groups were there any significant effects of the cycling sprint on the IL-10 concentration (*P* > 0.05) (Table 4).

### Comparison of Adipocytokine and Interleukin Level Between Sexes

Before the exercise, leptin plasma concentration in women totaled 7.09 ± 3.93 ng/mL and was higher (*P* < 0.001) than in men (1.27 ± 1.02 ng/mL). The concentrations of other biochemical markers before exercise were comparable in both groups (*P* > 0.05) (Tables 3, 4).

The concentration of irisin in the blood plasma of women was significantly higher (*P* < 0.05) than in men 15′, 30′, and 60′ after the exercise (Table 3).

Leptin concentration was significantly higher (*P* < 0.05) in women for all measurements after the exercise (3′, 15′, 30′, 60′, and 24 h) (Table 3).

IL-6 concentration in the serum at 60′ after exercise was higher (*P* < 0.05) in women than in men. There were no significant differences between sexes in IL-1β and IL-10 concentrations (Table 4).

### Correlations

A positive correlation was found between the resting concentration of asprosin and BM (*r* = 0.45, *P* = 0.049), as well as the negative correlation between resting leptin concentration and BM (*r* = -0.46, *P* = 0.04), LBM (*r* = -0.61, *P* = 0.004) and a positive correlation of leptin concentration with %FAT (*r* = 0.74, *P* < 0.001). There was a simultaneous, negative correlation between leptin concentration and MAP (*r* = -0.70, *P* = 0.001) and MP (*r* = -0.67, *P* = 0.001).

**FIGURE 1:**
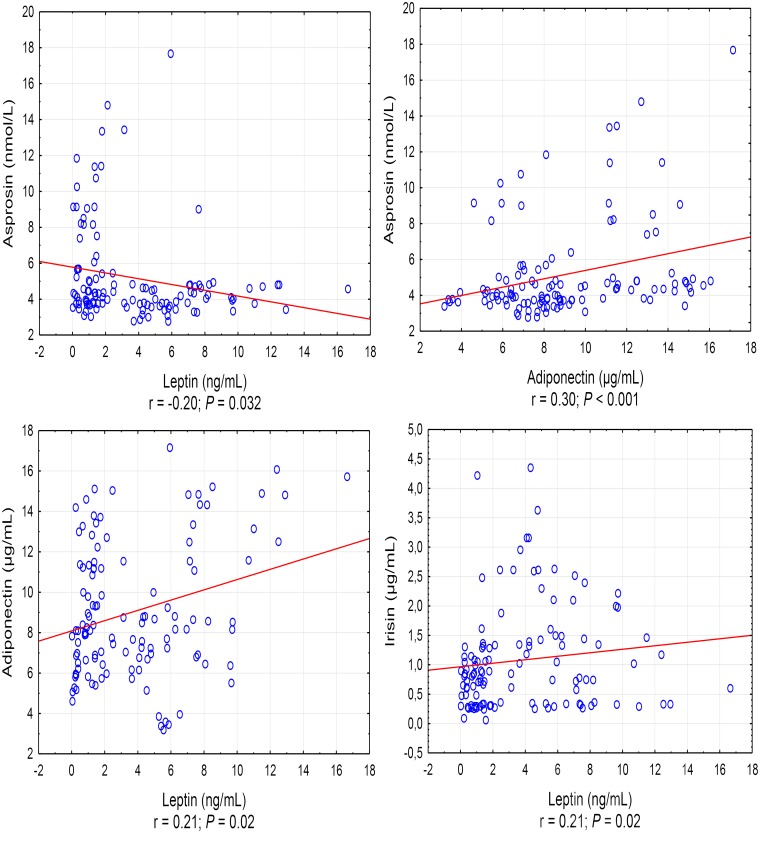
Statistically significant (*P* < 0.05) correlations between the exercise induced level of adipocytokine. *r* – correlation coefficient.

**FIGURE 2:**
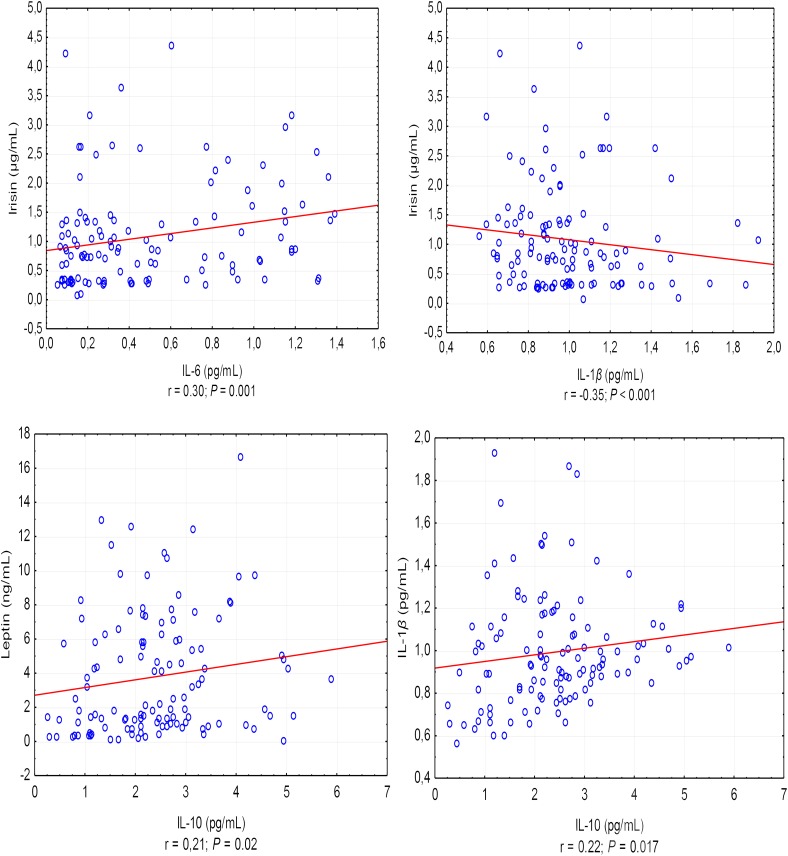
Correlations between the exercise-induced level of IL-6 and IL-1β, and the exercise-induced level of irisin and between the exercise-induced level of IL-10 and the exercise-induced level of leptin and IL-1β. *r* – correlation coefficient, *P* < 0.05 – level of statistical significance.

A positive correlation was found between the asprosin exercise-induced and adiponectin concentrations (*r* = 0.30, *P* < 0.001), and a negative correlation between asprosin and leptin concentration (*r* = -0.20, *P* = 0.032) (Figure 1). It was also shown that leptin exercise-induced concentration correlates positively with the concentration of adiponectin, irisin, and IL-10 (in each case, *r* = 0.21, *P* = 0.02) (Figure 1). Exercise-induced IL-6 concentration correlated positively with irisin concentration (*r* = 0.30, *P* = 0.001) and negatively with IL-1β concentration (*r* = -0.35, *P* < 0.001), while IL-1β concentration showed a positive correlation with IL-10 concentration (*r* = 0.22, *P* = 0.017) (Figure 2).

**FIGURE 3:**
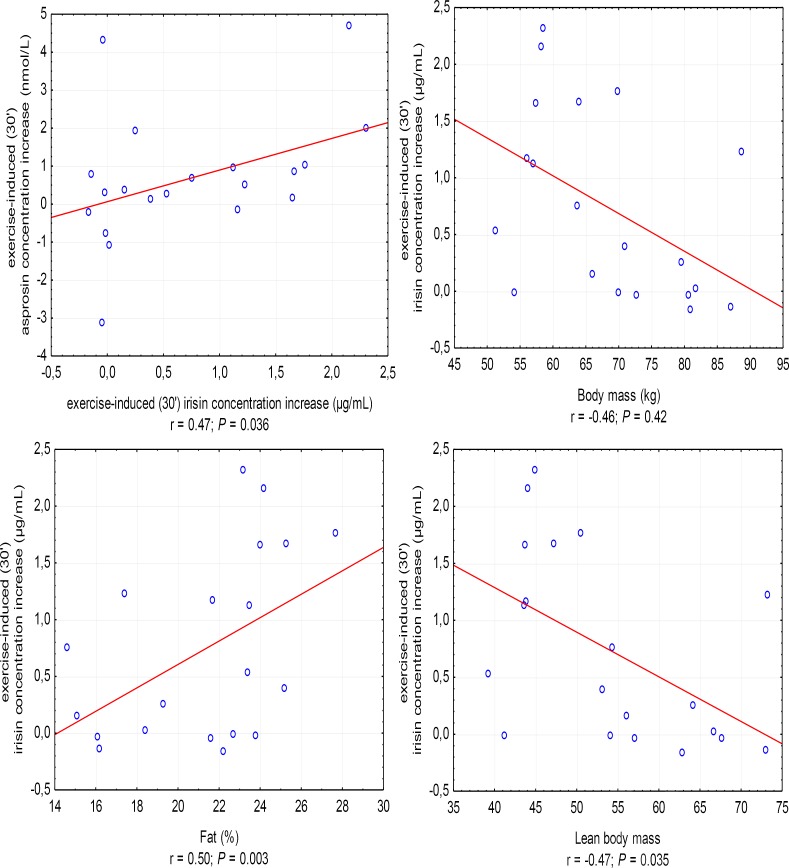
Correlations between the increase in irisin concentration in the 30′ following anaerobic exercise and the increase in asprosin concentration and body mass, fat content in percentages (%Fat), and lean body mass. *r* – correlation coefficient, *P* < 0.05 – level of statistical significance.

The increase in irisin concentration in the 30′ of recovery was positively correlated with the increase in asprosin concentration (*r* = 0.47, *P* = 0.036; in women, *r* = 0.67, *P* = 0.033) and %FAT (*r* = 0.50, *P* = 0.003), and negatively with BM (*r* = -0.46, *P* = 0.042) and LBM (*r* = -0.47, *P* = 0.035) (Figure 3).

## Discussion

Our study was the first to show that short-term anaerobic exercise is a factor inducing the secretion of asprosin and irisin, while the increase in blood levels of these cytokines only occurred in women. At the same time, anaerobic efforts in women caused a decrease in blood leptin levels. We did not find this effect in men. Regardless of sex, the effect of anaerobic exercise was an increase in IL-6 concentration and, at the same time, a decrease in IL-1β concentration in the blood. Following anaerobic exercise, there were no changes in the levels of adiponectin or IL-10, anti-inflammatory cytokines in any of the groups.

In our research, we used a 20-s bicycle sprint as the test effort. This is an exercise during which the anaerobic metabolism of glucose derived directly from muscle glycogen and the uptake of blood from myocytes prevails. ATP resynthesis during such an exercise is mainly the result of the substrate phosphorylation process. Anaerobic glycolysis, as a result of which pyruvic acid is finally reduced to lactic acid, causes a significant increase in blood lactate and disturbances of acid–base balance (Beneke et al., 2002; Wiecek et al., 2017). In our research, both groups showed a significant increase in blood lactate after exercise, but only in the group of women did the blood glucose level decrease. Considering that asprosin causes the release of glucose from the liver cells, and its “exclusion” is a significant impairment of this process (Romere et al., 2016), the blood glucose levels in women after anaerobic exercise may be a factor inducing its secretion. The lowest glucose level coincided with the highest concentration of asprosin in the blood (during the 30′of recovery).

Asprosin is a fasting-induced glucogenic hormone (Romere et al., 2016). It has been proven that circulating asprosin crosses the blood–brain barrier and activates orexigenic AgRP^+^ neurons *via* the cAMP dependent pathway, affecting appetite (Duerrschmid et al., 2017). The subjective sense of hunger immediately after the 30-s cycling sprint decreased and then increased, and during the 30′ after completing the effort, it was rated at about 49/100 points, although this study did not assess asprosin concentration (Bilski et al., 2016).

As in previous studies among subjects with normal glucose tolerance (Zhang et al., 2017), we did not find a correlation between blood asprosin and glucose concentrations, in contrast to the positive correlation that has been demonstrated in people with type 2 diabetes mellitus (T2DM) (Zhang et al., 2017). The concentration of asprosin in the case of people with T2DM and impaired glucose regulation was significantly higher than in healthy subjects (Wang et al., 2018) and positively correlated with body build indices such as waist circumference, waist–hip ratio (WHR) and BMI, as well as HbA1c, triglyceride, and HOMA-IR concentrations (Zhang et al., 2017; Wang et al., 2018). In the group of healthy subjects (Zhang et al., 2017), similarly as in our research, such correlations did not occur. In our study, however, we found a positive correlation with BM, without relation to the percentage fat content, which may suggest muscle tissue as a source of asprosin secretion, although we did not find a correlation between its concentration and LBM. At the same time, in our study, women were characterized by a significantly higher content of adipose tissue. *FBN1* expression in the skeletal muscle cells is known to be smaller than in subcutaneous and visceral adipose tissue adipocytes (Romere et al., 2016). Therefore, inference requires further research.

So far, the only study in which the concentration of asprosin in the blood related to physical exercise in humans was the experiment by Schumann et al. (2017), the results of which have been published as a post-conference report. In this study, it was found that the resting level of asprosin is comparable in obese women and men, at the same time, it is close to the level for non-obese subjects, but with very large differences of results within groups. Also, an acute bout of treadmill exercise (graded effort) did not affect asprosin secretion in obese and lean subjects (Schumann et al., 2017).

Despite the fact that the anaerobic effort caused significant changes in adipocytokine blood levels only in women, in our research, we showed that regardless of sex, the anaerobic-induced secretion of asprosin is associated with the secretion of other adipocytokines. At the same time, a positive correlation between asprosin concentration and adiponectin level, and negative correlation with leptin concentration, indicates the positive, anti-inflammatory effect of this hormone among young people with normal body composition. Upregulation of ATP resynthesis during the sprint leads to an increase in the availability of AMP (increase in the AMP/ATP ratio) (Morales-Alamo and Calbet, 2014), which may affect the secretion of irisin. This suggests the participation of irisin in the regulation of energy balance of the cell mediated by AMP-activated protein kinase (AMPK), the activity of which increases during anaerobic exercise (Guerra et al., 2010; Fuentes et al., 2012).

In our study, the exercise-induced increase in asprosin concentration during the 30′of recovery, as compared to the 3′ after exercise, was positively correlated with the increase in irisin concentration. Differences in irisin concentrations as a response to physical exercise may reflect the energy status of muscles during and after exercise. It has been proven that in less trained people, the concentration of irisin significantly increases during exercise, and in people training regularly, this increase is smaller (Huh et al., 2012). This is indirectly confirmed by our research. The effect of anaerobic exercise was increased secretion of irisin only in the group of women who had lower physical fitness capacity (aerobic and anaerobic) than men. The exercise-induced increase in blood levels of irisin among women was also associated with gender differences in body composition, because it positively correlated with body fat and negatively, with lean and total BM.

It was found that aerobic efforts performed on a regular basis induce increased secretion of adiponectin (Kriketos et al., 2004; Fatouros et al., 2005; Rashidlamir and Saadatnia, 2012). Strength training has shown an increase (Fatouros et al., 2005) or no changes in adiponectin levels (Klimcakova et al., 2006). In our research, neither in men nor women was anaerobic exercise a factor inducing changes regarding adiponectin levels in the blood. However, the research by Avazpor et al. (2016) showed that high-intensity interval training (HIIT), using two different models applying short running efforts with an intensity of over 90% HRmax, implemented for 8 weeks, caused a significant increase in adiponectin levels in the blood of young women, and at the same time, lowering leptin concentration. Leptin is produced not also by adipocytes but also the skeletal muscles and other tissues (Gnacinska et al., 2009). The leptin concentration, in contrast to adiponectin, positively correlates with the mass of adipose tissue (Fernandez-Sanchez et al., 2011). In our group of volunteers, the concentration of leptin positively correlated with the percentage of fat, negatively with total as well as LBM and was higher in women.

In research concerning a group of men and women with normal body composition, there were significant individual differences in leptin reaction (increase in level, decrease, or no change) to motor therapy of varying intensity (Perusse et al., 1997). In turn, no changes were observed in leptin levels or as a result of single efforts, regardless of their intensity (Kraemer et al., 2003; Kyriazis et al., 2007). However, a significant decrease in blood leptin concentration was noted 24 h after a single endurance exercise in women and men with type 2 diabetes (Kanaley et al., 2001). In our study among men, as well as after a single 30-s bicycle sprint in the trial by Bilski et al. (2016), the concentration of leptin did not change in the blood, while in women, it decreased already during the 3′ following exercise, and was reduced by 30 min of rest. In other studies (Guerra et al., 2011) among men, the reduction of blood leptin levels occurred only 4 h after the end of the cycling sprint. Guerra et al. (2011) showed that despite the lack of changes or a slight reduction in blood leptin levels, a bicycle sprint, at various times after exercise (30–120 min), increases the activity of many leptin-activated signaling pathways. The content of PGC-1α mRNA in the m. vastus lateralis cells, increased about 3.2-fold 4 h after exercise (Guerra et al., 2011). These results (Guerra et al., 2011) suggest that even a single sprint is able to activate the same signaling cascades as leptin, and therefore, this type of effort could be used as a leptin mimetic to circumvent leptin resistance (or in the HIIT models) in the anti-inflammatory therapy for obesity or diabetes.

Our research also points to the anti-inflammatory effect of anaerobic exercise. We have found that the bicycle-sprint sprout induced an increase in serum IL-6 concentration, at the same time associated with a decrease in pro-inflammatory IL-1β concentration. This effect of exercise was independent of gender, and the correlation between IL-6 and IL-1β concentration was negative, which in this case, demonstrates the anti-inflammatory function of IL-6 (Zembron-Lacny and Ostapiuk-Karolczuk, 2008; Donma and Donma, 2018). Increased IL-6 concentration in the blood following a cycling sprint was also demonstrated by previous studies (Guerra et al., 2011), although changes in levels occurred only after 4 h of recovery, contrary to our results, where the IL-6 concentration was elevated in the 3′-60′ range of recovery among men, and from 15′ to 60′ of rest, as well as 24 h after completing the sprint in women. At the same time, in our research, the concentration of IL-6 was positively correlated with the concentration of irisin, which, at the proper level, is also ascribed to anti-inflammatory activity (Mazur-Bialy et al., 2017; Donma and Donma, 2018).

## Conclusion

In healthy, young individuals, a single anaerobic exercise increases asprosin and irisin secretion and reduces leptin secretion in women. Changes in the concentration of adipocytokines are inter-related. Regardless of sex, anaerobic efforts induce anti-inflammatory effects.

However, further research is required on the influence of physical efforts of varying intensity on the secretion of asprosin and other adipocytokines/myokines. These trials should involve more subjects, including obese individuals.

## Author Contributions

MW conceived the project and procured the project funding. MW, JS, MM, and ZS contributed to the collection of the data and reagents. MK developed and analyzed diets. MW performed the statistical analysis and interpretation of results. MW drafted the first version of the manuscript. ZS conducted substantive consultations. All authors contributed to revising the manuscript and gave their final approval of the submitted version.

## Conflict of Interest Statement

The authors declare that the research was conducted in the absence of any commercial or financial relationships that could be construed as a potential conflict of interest.
